# Network Pharmacology-Based Validation of the Efficacy of *Huiyangjiuji* Decoction in the Treatment of Experimental Colitis

**DOI:** 10.3389/fphar.2021.666432

**Published:** 2021-05-28

**Authors:** Wei Yu, Hongju Cheng, Baoliang Zhu, Jing Yan

**Affiliations:** Department of Physiology, Jining Medical University, Jining, China

**Keywords:** inflammatory bowel disease, macrophages, intestinal organoids, *Huiyangjiuji* decoction, ulcerative colitis

## Abstract

Ulcerative colitis (UC) is the major type of inflammatory bowel disease (IBD) characterized by an overactive immune responses and destruction of the colorectal epithelium with intricate pathological factors. In China, *Huiyangjiuji* decoction (HYJJ) has been widely administered against inflammation, but the underlying mechanical mechanisms are not known. A murine model of colitis was established by orally feeding 4% dextran sodium sulfate for 5 days. Intestinal organoids (IOs) were treated with TNFα (Tumor necrosis factor-α) as an *ex-vivo* UC model. A scratch assay combined with a co-culture system that incubated murine epithelial cell line (IEC-6) with macrophages (Mφs) was utilized to assess epithelial recovery under inflammatory conditions. Network pharmacology analysis was performed to elucidate the mechanism of HYJJ decoction. In the present study, we confirmed that HYJJ considerably alleviated of DSS-induced colitis, as evidenced by the improved intestinal injury and fecal albumin, as well as feces blood. Network pharmacology analysis identified the active components in HYJJ formula, and KEGG enrichment analysis indicated that HYJJ-target genes were enriched in pathogen-induced infections, cancer-related as well as inflammatory pathways. Consistently, RNA-sequencing demonstrated that HYJJ treated inhibited cytokine-cytokine interaction, IBD as well as TNF signaling pathways, confirming the anti-inflammatory and anti-neoplastic role of HYJJ decoction. *In-vitro* experimental evidence confirmed the suppression of pro-interleukins by HYJJ, including IL-2, IL-10 and IL-12. Moreover, the contribution of HYJJ to mucosal healing was corroborated by *ex-vivo* experiments, in which HYJJ rescued TNFα-compromised IOs functions, i.e., elevated mitochondrial stress (MOS) and impaired regeneration capacity. IEC-6 cells co-culture with Mφs from HYJJ-treated experimental colitis mice showed an improved migration capacity as compared to those incubated with Mφs from untreated colitis mice. We conclude that HYJJ re-establishes homeostasis of the gut epithelium during colitis by suppressing inflammation and orchestrating cytokines interaction.

## Introduction

Traditional Chinese Medicine (TCM) has aroused great attention due to the abundance of multiple compounds with proven clinical efficacy that have multitarget properties. However, there are concerns worldwide regarding the efficacy and toxicity of TCM due to inadequate pharmacological evidence. Given that efficacy and toxicity are always the major sources of attrition in TCM, network pharmacology provides the promise of elucidating the mechanical mechanisms of TCM by a more holistic approach for integrating compound-target interactions from a molecular to system level.

It has long been known that the pathogenesis of Inflammatory bowel disease (IBD) involves a broad spectrum of factors. In addition to the contribution of genetic variants to the heterogeneity of IBD (Voskuil et al., 2020), physiological environmental dimension, e.g., ingestion and psychological condition also influence the progression (van der Giessen et al., 2020; Borren et al., 2020). Moreover, these different factors are interlinked and regulate gut microbiome and host-microbiota interaction, hence acting on modulation of IBD pathogenesis (Jazayeri et al., 2017). As of today, the pathogenesis remains still a matter of discussion due to the unpredictable and heterogeneous clinical course of IBD (Voskuil et al., 2020). IBD is pathologically characterized by epithelium disruption under a sustained pro-inflammatory microenvironment, and has two main forms-Crohn’s disease (CD) and Ulcerative colitis (UC). CD can induce lesions alongside all the gastrointestinal system, i.e., from mouth to anus, whereas UC is generally limited to the colon (Veauthier and Hornecker, 2018). UC is an independent risk factor for the progression of colorectal cancer (CRC) (Xue et al., 2018), and UC-associated CRC has a much poorer survival probability as compared to sporadic CRC (Jess et al., 2006). Mucosal healing, one primary concern when developing a potential therapeutic target for UC, requires homeostasis of the intestinal stem cells (ISCs) niche. Any disruption in the ISC niche will destroy the renewal of epithelium, thereby causing gastrointestinal disease (Merlos-Suárez et al., 2011; Suzuki et al., 2018; Yu et al., 2020). Herewith, establishing an anti-inflammatory microenvironment in favor of ISC-initiated wound healing and restoring healthy gut function would be an ideal therapeutic strategy, and thus the multitarget nature of TCM would shed light on UC therapy in the clinic.

*Huiyangjiuji* decoction (HYJJ) formula from Shang Han Lun that is the oldest Chinese monograph on Cold Damage Diseases (Chen et al., 2009), encompasses *Aconitum carmichaelii debx* (ACD), *Zingiberis Rhizoma* (ZR), *Panax Ginseng C. A. Mey* (PGM)*, Glycyrrhiza uralensis Fisch* (GF)*, Atractylodes Macrocephala Koidz* (AMK), *Cinnanmomi Cortex* (CC), *Citrus Reticulata* (CR), *Schisandrae Chinensis Fructus* (SCF), *Poria Cocos (Schw.) Wolf* (PCW)*, Pinellia ternate* (Thunb.) Breit (PB)*.* Among these herbs, the combination of ACD, ZR and GF also termed by Si-Ni decoction has shown proven efficacy for septic shock (Chen et al., 2011), sepsis (Brislinger et al., 2018; Zeng et al., 2019) by interfering with apoptosis and inflammatory responses (Aly et al., 2005; Endo et al., 2017; Yang et al., 2017; Endo et al., 2018; Bai et al., 2020). In the context of SI-NI decoction, the anti-inflammatory function has been emphasized by the addition of PGM (Jin et al., 2020), PB (Tian et al., 2020), SCF (Panossian & Wikman, 2008; Li et al., 2020; Li et al., 2021), PCW (Zhang et al., 2018) as well as CC (Csikos et al., 2020). Moreover, focusing on the impaired gastrointestinal functions, AMK is included to improve gastrointestinal motility (Zhu et al., 2018; Yang et al., 2019) and restore immune homeostasis (Xiang et al., 2020), while CR maintains homeostasis of the gut microbiota (Chen et al., 2020) and inhibits inflammation (Ho & Lin, 2008; Herath et al., 2016). Combining various anti-inflammatory herbs with gut-friendly components has offered a promising therapeutic alternative for gut diseases, especially for UC and UC-CRC.

In the present study, we discussed the potential therapeutic effect of HYJJ in UC by network pharmacology combined with RNA-sequencing, and examined the influence of HYJJ *in vivo* and *in vitro*.

## Materials and Methods

### Ethics Statement

All procedures and assays were approved by the Institutional Animal Care and Use Committee of Jining Medical University.

### Screening of Active Ingredients and Targeted Genes in *Huiyangjiuji* Decoction Components

We screened the active components according to Oral bioavailability (OB) values and drug-likeness (DL) utilizing Traditional Chinese Medicine Systems Pharmacology Database and Analysis Platform (TCMSP) (Ru et al., 2014). The ingredients greater or equal to 0.1 (Drug likeness, DL) and 20% (OB) were included according to TCMSP parameter information and criteria.

The targets of each active ingredient in HYJJ were retrieved from TSMSP database and transformed into gene symbols of *Homo sapiens* species by the UniProt knowledge database (www.uniport.org).

### Inflammatory Bowel Disease-Related Genes Acquisition

We collected IBD-related genes from five sources with the keyword “ulcerative colitis,” including GeneCards (Rebhan et al., 1997; Safran et al., 2010), DrugBank (Wishart et al., 2018), Online Mendelian Inheritance in Man (OMIM) (Hamosh et al., 2002), PharmGkb (Whirl-Carrillo et al., 2012), and Statistics of Therapeutic Target Database (TTD) (Wang et al., 2020) (Supplementary Table S1).

### Establishment of the Herb–Ingredient–Target Interaction Network

We intersected the targeted genes of HYJJ decoction with disease genes (Supplementary Table S4), and constructed a Venn diagram. By Cytoscape software (Su et al., 2014) Herb–Ingredient–Target (HIT) interaction network was visualized.

### Protein-Protein-Interaction Network Construction

STRING database (Search Tool for Retrieval of Interacting Genes/Proteins) (http://string-db.org/) (Szklarczyk et al., 2019) was used to construct a protein-protein interaction (PPI) network based on the obtained HIT gene symbols. Full PPI network with both functional and physical protein associations and without disconnected nodes was constructed with a confidence score of ≥0.7 for significance.

Network nodes represent proteins, and edges represent protein-protein associations that proteins jointly contribute to a shared function, but this does not mean they are physically binding each other. The thicker of edge means the higher edge confidence.

### Hub-Genes Identification

Hub genes with a high-degree node were identified by Cytocape plugin CytoNCA according to betweenness centrality (BC), closeness centrality (CC), degree centrality (DC), eigenvector centrality (EC), local average connectivity (LAC) and network centrality (NC) (Tang et al., 2015). These features were calculated twice to establish the final sub-network consisting of hub-genes with higher than the median values of the features.

### GO and KEGG Pathway Enrichment

GO (Ashburner et al., 2000) and KEGG enrichment analysis (Kanehisa and Goto, 2000) of genes were implemented by Bioconductor (R). GO terms with adjusted *p*-value below 0.05 were considered as significantly enriched genes.

### Experimental Validation

#### Animal

C57BL/6 mice were purchased (Cyagen, China). All animals were housed at sperm free animal facility at Jining medical university. All procedures involving animals were conducted within IACUC guidelines under approved protocols.

#### Ulcerative Colitis Mouse Model Subjected to *Huiyangjiuji* Decoction Administration

ACD, ZR, PAM, GF, AMK, CC, CR, SCF, PCW, PB were mixed according to the ratio of 3:2:2:3:3:1:2:1:3:3. The mixture was added to 1000 ml (1:10 g/v) of pure water and boiled for 1 h.

A total of 18 mice were randomly divided into three groups (6 per group): control group, DSS group, HYJJ-treated DSS group. 4% dextran sodium sulfate (DSS, MP Biomedicals) was added to drinking water for 4 days. Upon removal on day 5, HYJJ decoction solution was administered for 1 week. UC was scored by the following standards: i) weight loss (no loss = 0; <5% = 1; 5–10% = 2; 10–20% = 3; >20% = 4); ii) stool (normal = 0; soft, watery = 1; very soft, semi-formed = 2; liquid, sticky, or unable to defecate = 3); iii) bloody stool test (no blood test positive within 2 min = 0; purple positive after 10 s = 1; light purple positive within 10 s = 2; heavy purple positive within 10 s = 3) (Leagene); iv) Histological injury and inflammation were scored by parameters including edema, destruction of the epithelial monolayer, crypt loss, infiltration of immune cells into the mucosa.

Tissues were fixed and then embedded in paraffin. 3 μm slice was sections stained with hematoxylin and eosin (H&E).

#### Identification of the Chemical Constituents of *Huiyangjiuji* Decoction

Hybrid Quadrupole-TOF LC/MS/MS Mass Spectrometer was utilized. 0.1 g decoction powder was added to 1 ml 80% methanol under ultrasonication for 15 min. After 1200 r/min centrifugation for 10 min, the filtrate was collected through a 0.45 μm membrane filter and injected into Hybrid Quadrupole time-of-flight LC/MS/MS Mass Spectrometer (Triple TOF 5600+, AB Sciex Instruments) to identify the chemical constituents.

Chemical identification was performed on a connected system of LC-30 (Shimadzu)-Hybrid Quadruple time-of-flight mass spectrometer (TOF MS) with electrospray ionization source (ESI). InerSustain C18 column (Shimadzu, 100 mm × 2.1 mm, 2 µm) was used to perform chromatographic separation with a flow rate of 0.3 ml/min at 35°C. A linear gradient program with mobile phase system including Equate A (acetonitrile) and Equate B (0.1% HCOOH-H_2_O): 4 min (A:5%:B:95%), 8 min (A:20%:B:80%), 2 min (A:15%:B:75%), 2 min (A:46%:B:54%), 3 min (A:100%:B:0%), 1 min (A:5%:B:95%).

Following is the instrumental settings: both ion source gas 1 and gas 2 were 50 psi, curtain gas (CUR) was 25 psi, source temperature was 500°C in positive mode while 450°C in negative mode, ion spray voltage floating (ISVF) was 5500 V in positive mode while 4400 V in negative mode, TOF MS scan range was 100–1200 Da, product ion scan range was 50–1000 Da, TOF MS scan and product ion scan accumulation time were 0.2 and 0.01 s, respectively. Data was acquired in information-dependent acquisition (IDA) with high sensitivity mode, collision energy was 35 ± 15 eV, and declustering potential was ±60 V (Supplementary Table S2; Supplementary Figure S1).

#### *Huiyangjiuji* Decoction Solution Preparation

In a bid to prepare HYJJ solution for *in vitro* experiments, we established a HIT interaction network with the hub genes and the active components of herbs (Supplementary Figure S2). We selected the shared components of these herbs, included one typical ingredient with the highest degree in each herb: PCW (Hederagenin); AMK(3β-acetoxyatractylone), ACD (Karanjin), CC (Oleic acid), CR (Nobiletin, Naringenin), PCM (Kaempferol, beta-sitosterol), PB (Stigmasterol, Baicalein), SCF (Gomisin R), ZR (Sexangularetin), GF (Quercetin). MTT assay was utilized to ascertain toxicity of the mixture in DLD1 cells (Human colorectal cell line) (Supplementary Figure S3). These selected chemicals were mixed at a concentration of 10 ng/ml of each chemical diluted in DMSO (All chemicals have purchased the chemicals in Yuanye Biology, China).

#### Ribonucleic Acid Sequencing

We treated DLD-1 cells withTNFα (30 ng/ml) (Stemcell technology) for 24 h and added HYJJ mixture. After 14 h, RNA was exacted for the experiment. RNA degradation and contamination was monitored on agarose gels. RNA purity, concentration and integrity were checked using the spectrophotometer, Qubit and the Agilent 2,100, respectively. A total amount of 2 µg RNA per sample was used as input material for the RNA sample preparations. Sequencing libraries were generated using VAHTS mRNA-seq v2 Library Prep Kit for Illumina following manufacturer’s recommendations and index codes were added to attribute sequences to each sample. Briefly, mRNA was purified from total RNA using poly-T oligo-attached magnetic beads. Fragmentation was carried out using fragmentation buffer. First strand cDNA was synthesized and second strand cDNA synthesis was subsequently performed. Remaining overhangs were converted into blunt ends. After adenylation of 3′ ends of DNA fragments, adaptor with hairpin loop structure were ligated. Then the PCR was performed. At last, Qubit HS quantification, Agilent 2,100 Bioanalyzer/Fragment Analyzer 5,300 quality control, the final library size of about 350 bp. The libraries were sequenced on an Illumina NovaSeq platform to generate 150 bp paired-end reads, according to the manufacturer’s instructions.

Raw data (raw reads) of fastq format were firstly processed through primary quality control. In this step, clean data (clean reads) were obtained by removing read pairs that contain N more than three or the proportion of base with quality value below five is more than 20%, in any end, or adapter sequence was founded. All the downstream analyses were based on the clean data with high quality. Paired-end clean reads were aligned to the reference genome using TopHat. We selected TopHat as the mapping tool for that TopHat can generate a database of splice junctions based on the gene model annotation file and thus a better mapping result than other non-splice mapping tools. Quantification of gene expression level HTSeq was used to count the reads numbers mapped to each gene. Identification of differentially expressed genes Differential expression analysis between two conditions was performed using the DEGSeq R package (1.20.0). Differentially expressed genes were defined as those for which the adjusted *p*-value below 0.05 and the log2 (Fold change) more than 1. Raw data has been submitted to SRA database (PRJNA724825).

#### Peritoneal Macrophages Isolation

Mice were sacrificed and cold PBS was injected. Peritoneal liquid was collected and centrifuged. The pallet was diluted with RPMI1640 medium.

#### Intestinal Organoids Culture

MIOs were isolated and cultured according to the protocol (Stemcell technology). Small intestine was washed with cold PBS for 15 times and digested (Stemcell technology). The supernatant was filtered and centrifugated at 1300 rpm for 5 min. The pellet was cultured in intestiCult organoid growth medium (Stemcell technology). The medium was exchanged every 2 days.

#### Immunofluorescence

For immunofluorescence, intestinal organoids were incubated with MitoSOX™ Red Mitochondrial Superoxide Indicator (ThermoFisher) for 10 min at 37°C. After PBS washing, cells were mounted on the fluorescent scope at 590 nm.

#### Western Blotting

Cells were lyzed and the extracts were centrifuged at 13,000 rpm for 20 min at 4°C. Total protein (40 µg) was subjected to 8% SDS-PAGE. Proteins were transferred to a nitrocellulose membrane (VWR) and the membranes were then blocked overnight at 4°C. Antibody included BAX, BCL-2 and GAPDH antibodies (1:1000) (ThermoFisher). After secondary antibody incubation, protein bands were visualized after anti-rabbit IgG conjugated to horseradish peroxidase treatment.

#### Scratch Assay

Intestinal epithelial cells-6 (IEC-6) cells were seeded and the monolayer was scratched. The scratched area is photographically monitored at 0 and 24 h after treatment. The percentage of the coverage was measured.

#### Enzyme-Linked Immunosorbent Assay

Mouse IL-2, IL-12, IL-10 as well as Mouse Albumin ELISA Kit were purchased from Abcam. Serum was incubated with antibody cocktail for 1 h. After washing for three times, the supernatant was discarded. Streptacidin-HRP solution was added and incubated for 1 h. Then TMB solution was added and incubated for 10 min. After stop solution was utilized, the plate was mounted and OD was read at 450 nm.

#### Statistics

Data are provided as means ± SEM, n represents the number of independent experiments. All data were tested for significance using Student’s unpaired two-tailed t-test and only results with *p* < 0.05 were considered statistically significant.

## Results

### Huiyangjiuji Decoction Alleviated Ulcerative Colitis Progression

**FIGURE 1:**
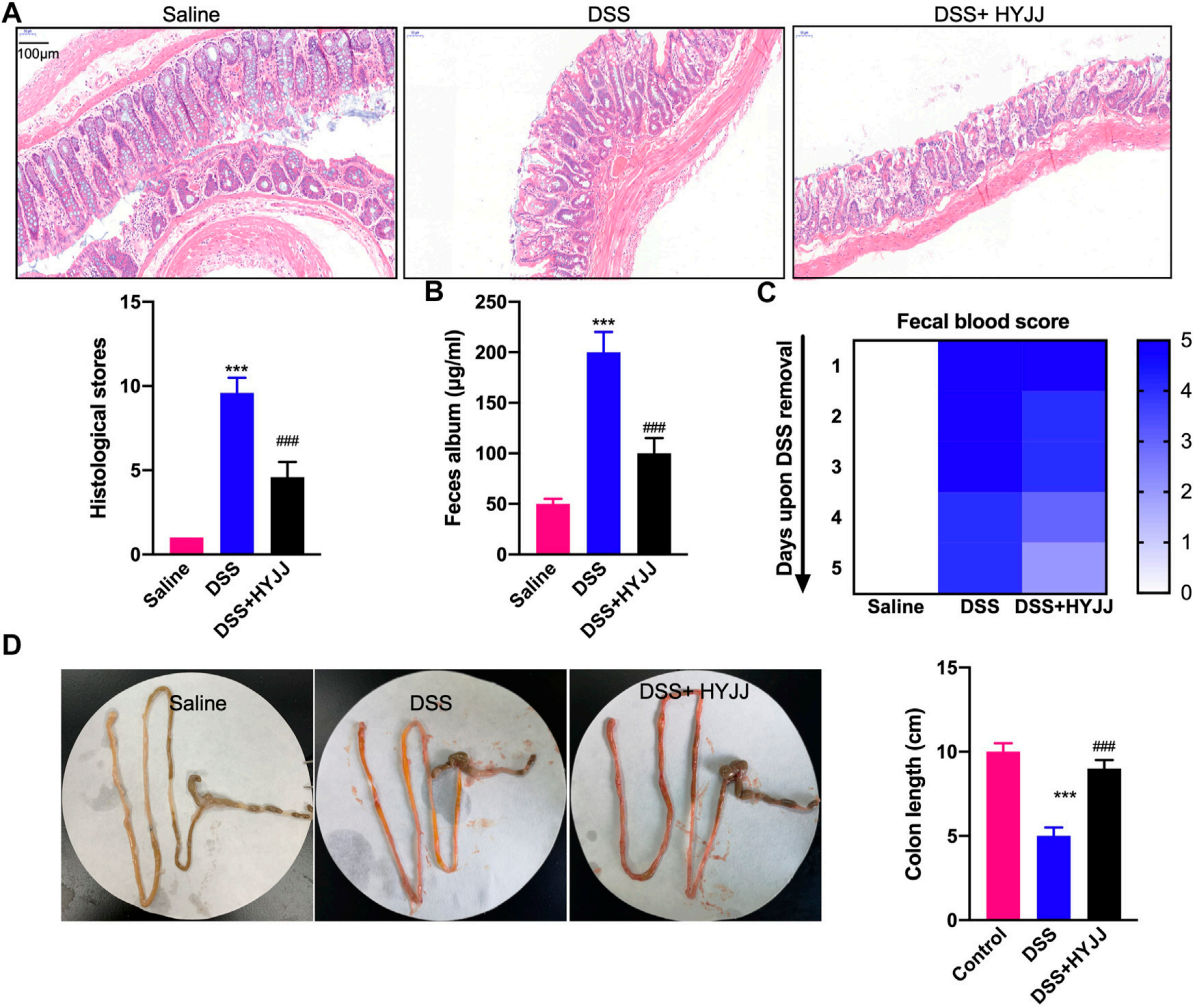
HYJJ alleviates UC progression. **(A)** Representative HE staining images of duodenum and colon sections from C57 mice subject to 4% DSS administration with HYJJ at **5** mg/kg every 2 days. Scale bar, 150 μm. (B) Albumin level of IBD mice with HYJJ. **(C)** Fecal occult blood test of IBD mice with HYJJ.**(D)** Small intestine and colon from control and IBD mice with HYJJ therapy. ***(*p* < 0.001) indicate statistically significant difference from saline group, ###(*p* < 0.001) indicate statistically significant difference from IBD group.

A murine model of ulcerative colitis was established by feeding 4% DSS water to C57 male mice weighing 20–25 g, six mice in a group with or without HYJJ administration. The severity of experimental colitis was defined according to epithelial deconstruction, shortened colon length, and fecal blood. As shown in Figure 1A, HE staining showed that HYJJ markedly alleviated the epithelial structural collapse in UC mouse models. Exacerbated fecal album and fecal blood in DSS group were apparently rescued after HYJJ treatment (Figures 1B,C). Consistently, HYJJ therapy successfully maintained the colon length of UC mouse models. Collectively, *in vivo* assay indicated that HYJJ could alleviate UC progression.

### The Herb-Ingredient-Target Network of Huiyangjiuji Decoction

W In a bid to ascertain the pharmacological mechanism of HYJJ, we screened the active components of HYJJ decoction in the TCMSP database and identified 188 active compounds targeting 2,165 genes*.* ACD yields 21 components targeting 92 genes, ZR yields five components targeting 54 genes*,* PCM*.* yields 22 components targeting 256 genes*,* GF yields 92 components targeting 1769 genes, AMK yields seven components targeting 23 genes, CR yields five components targeting 95 genes, SCF yields eight components targeting 30 genes, PCW yields 15 components targeting 30 genes, PB yields 13 components targeting 175 genes, CC yields 10 components targeting 116 genes (Supplementary Table S1).

**FIGURE 2:**
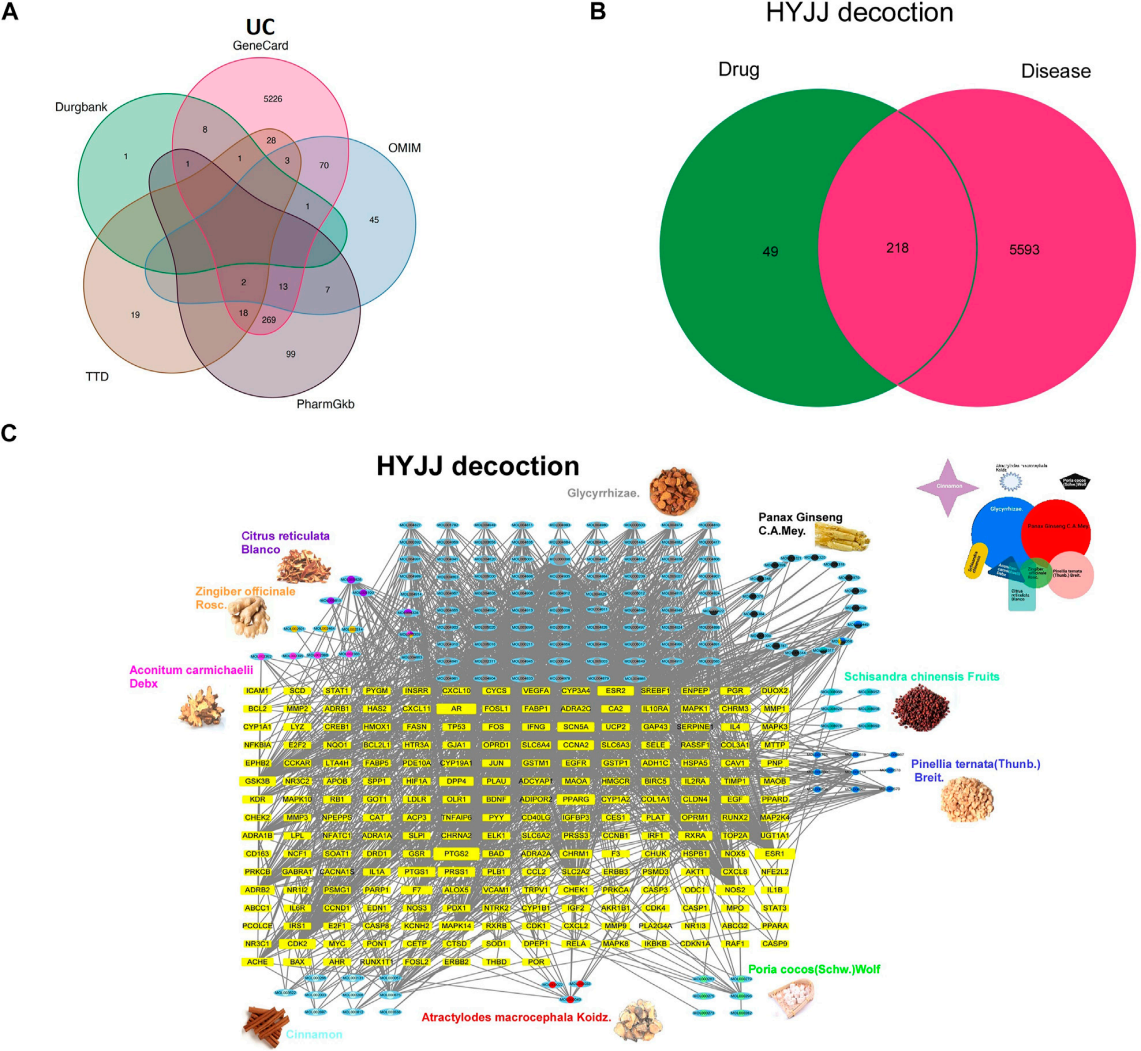
The Herb-Ingredient-Target network of HYJJ. A Venn analysis showing IBD-related genes from five databases: Drugbank, GeneCard, OMIN, PharmGkb, TTD. **(B)** Venn analysis showing the crosstalk between HYJJ and UC.**(C)** Herbs and compounds as well as all the potential targets were linked.

We screened five databases for UC-relevant genes and obtained 5,811 genes after deleting redundant items (Figure 2A, Supplementary Table S2). Venn diagram analysis further showed that HYJJ shared 218 putative targets with UC (Figure 2B). The overlapping gene symbols were detailed in Supplementary Table S3.

**FIGURE 3:**
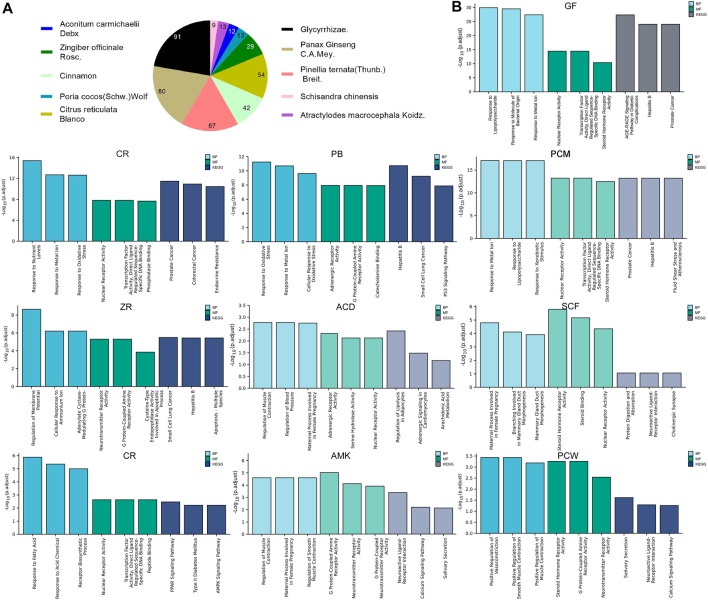
The function of each herb in the pathogenesis of UC. **(A)** Pie chart illustrating the target genes of each herb in UC.**(B)** GO and KEGG analysis showing the enrichment of herb-target genes.

To illustrate the pharmacological mechanism of HYJJ, we constructed a HIT network utilizing Cytoscape (Figure 2C). The yellow nodes represent UC genes and the blue nodes represent HYJJ molecules, while colorful dots within the component indicate different herbs, and the edges mean the interaction between UC and components. As illustrated, most herbs not only worked synergistically targeting the same genes, but shared some components, such as GR and ZR, PCM and SCF as well as PB. Furthermore, we explored the role of each herb in UC treatment. The pie chart showed the target genes of each herb in UC (Figure 3A), and GO and KEGG analysis was performed with these genes (Figure 3B). As the primary source of active components, GF and PCM target genes were similarily enriched in responses to lipopolysaccharide and bacterial as well as metal ions, steroid hormone pathway; CR and PB, as well as ZR influenced responses to oxidative stress and metal ions, while PB specifically regulated adrenergic and catecholamine activity and CR was involved in endocrine resistance; Of note was the anti-neoplastic role of the herbs mentioned above, indicating the potential therapeutic capacity of HYJJ in cancer handling. CR was responsible for responses to fatty acid and acid chemicals as well as the regulation of Adenosine 5‘-monophosphate (AMP)-activated protein kinase (AMPK) and peroxisome proliferators-activated receptors (PPAR) signaling pathways that exert an anti-inflammatory role in UC (Arafa et al., 2020; Xu et al., 2017). Other seemingly minor but vital herbs were ACD, SCF, PSW and AMK. Interestingly, ACD, AMK as well as PSW associated with muscle contraction and vasoconstriction, while AMK and PSW as well as SCF not only worked with neurological processes, including neuroactive ligand-receptor interaction and neurotransmitter receptor activity, but improved digestion processes, such as protein digestion and absorption, and salivary secretion. From the enrichment analysis angle, HYJJ decoction could suppress inflammation, orchestrate hormone activity and restore gut function, thereby holistically combating UC.

**FIGURE 4:**
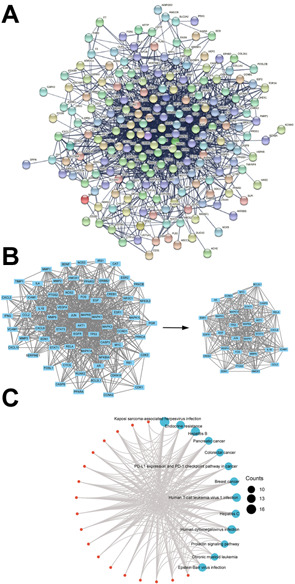
PPI network and hub genes. **(A)** PPI interaction network established by the target genes of HYJJ in UC. **(B)** Hub genes of PPI interaction network.**(C)** KEGG analysis of the hub genes.

We construct a full PPI network with a PPI enrichment *p*-value (<1.0 e-16) based on the STRING database (Figure 4A) and extracted 29 hub-genes (Figure 4B). The median values of BC, CC, DC, EC, LAC, and NC in the left subnetwork were 76.52928591, 0.4048583, 10, 0.030461067, 5.333333333, and 6.5, respectively. The median values in the right subnetwork (hub genes) were 15.67041167, 0.591562335, 19.5, 0.1026759405, 11.64171123, 13.451165665, respectively. 29 hub genes with higher than the median values of the features were identified (Figure 4B). In line with the individual function of each herb, KEGG analysis demonstrated that the hub-genes in the HIT network were enriched in infection, cancer and endocrine processes.

### Expression Profile of Human Colorectal Cancer Cells With Huiyangjiuji Decoction Treatment

**FIGURE 5:**
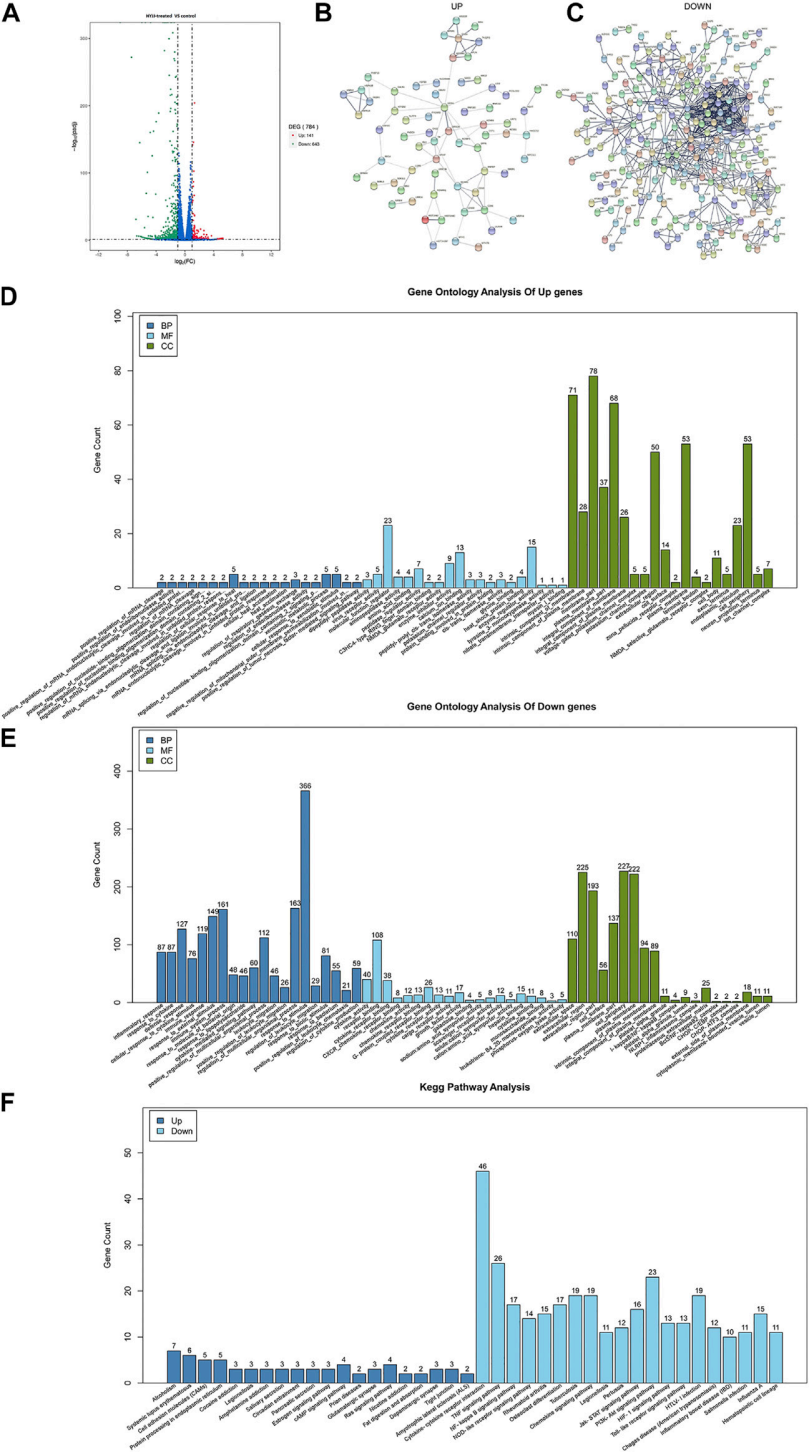
Gene Ontology (GO) and KEGG enrichment analysis of the targets. **(A)** Chart illustrating the DEGs between control and HYJJ-treated DLD-1 cells. **(B–C)** PPI interaction network established by up-regulated genes or down-regulated genes. **(D)** GO analysis of up-regulated genes of HYJJ treated DLD-1 cells. **(E)** GO analysis of Down-regulated genes of HYJJ treated DLD-1 cells. **(F)** KEGG analysis of DEGs of HYJJ treated DLD-1 cells.

Network pharmacological analysis not only indicated the anti-inflammatory effect of HYJJ, but also pointed out the anti-neoplastic potential of this decoction. Given that UC has been regarded as an independent risk factor of UC-CRC, we treated DLD-1 cells with HYJJ and extracted RNA for RNA-sequencing. As a result, 784 DEGs were obtained between HYJJ group and the control group. Among these DEGs, 643 genes were down-regulated and 141 were up-regulated (Figure 5A). Based on the STRING database, PPI network composed of down-regulated or up-regulated genes were constructed (Figure B, and C). We then performed GO and pathway enrichment analysis. The top enrichments of up-regulated genes in CC were membrane part, intrinsic component as well as integral component of membrane; In BP were regulation of cell response to heat and xenobiotic stimulus; In MF were molecular function regulator and enzyme regulator activity. Of note, the top enrichment of down-regulated genes in CC was cell periphery, plasma membrane and extracellular region, in BP were response to stimulus, regulation of multicellular organismal process as well as inflammatory response, in MF were receptor binding and cytokine activity (Figure 5E). KEGG analysis showed that HYJJ treatment facilitated cell adhesion molecules, salivary and pancreatic secretion, glutamatergic and dopaminergic synapse, fat digestion and absorption, all of which were mentioned in network analysis about the individual role of AMK and PSW as well as SCF, these seemingly minor components of HYJJ. Not surprisingly, HYJJ down-regulated genes were enriched in inflammation-related pathways, such as cytokine-cytokine receptor interaction, TNF signaling pathway as well as IBD, NOD-like and Toll-like receptor signaling pathways (Figure 5F), fully corroborating the anti-inflammatory as well as the anti-cancer therapeutic role of HYJJ.

### Huiyangjiuji Decoction Rescued TNFα-Hampered TNFα-Hampered Intestinal Stem Cells Survival

**FIGURE 6:**
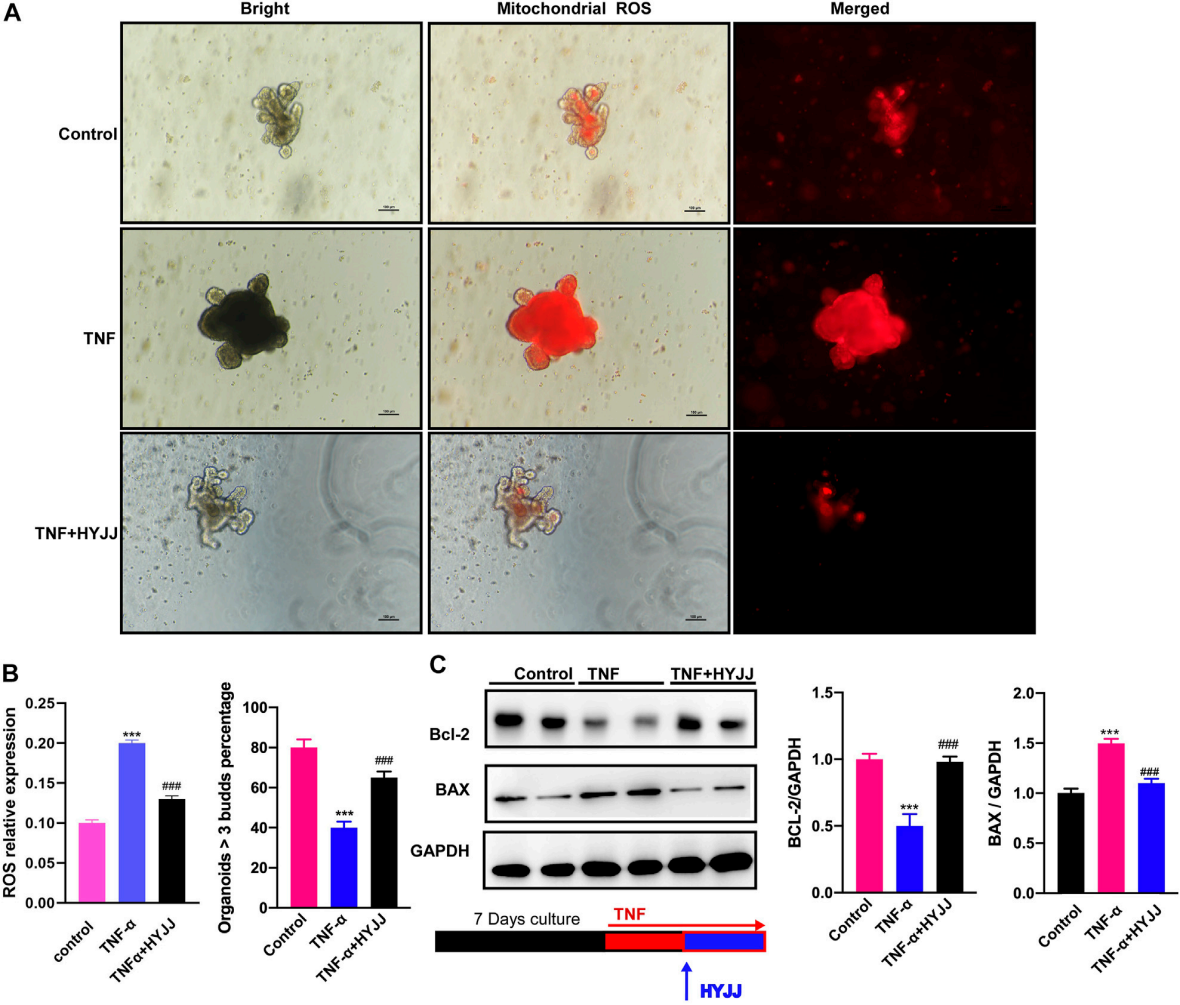
HYJJ rescued TNFα-hampered IOs functions.**(A)** Immunofluorescent staining illustrating the mitochondrial stress of TNFα-treated (20 ng/ml, 24 h) IOs in the presence of HYJJ (30 ng/ml, 24 h). **(B)** Bar charts (*n* = 5) illustrating the number of multi-buds IOs in the presence of HYJJ and TNFα. **(C)** Original western blot pictures and bar charts (*n* = 5) illustrating the protein expression of Bcl-2 and BAX in *α*-treated IOs with HYJJ. ***(*p* < 0.001) indicate statistically significant difference from saline group, ###(*p* < 0.001) indicate statistically significant difference from UC group.

Intestinal organoids (IOs) culture is an ideal tool to mimic the stem cell niche and investigate the stemness of ISCs under pathological settings. The *ex-vivo* IBD cellular model was established by treating IOs with TNFα (20 ng/ml, 24 h), and then HYJJ was administered for another 24 h. TNF is interconnected with ROS, dysregulation of which is a hallmark of cancers and inflammatory diseases (Blaser et al., 2016). Given the inhibition of TNF signaling pathway by HYJJ shown in RNA-seq results, and that excessive reactive oxygen species (ROS) enables stem cells to escape from a long-lived quiescent state-a slow-cycling state and enter into a proliferative state undergoing differentiation or apoptosis (Tothova et al., 2007; Zhang et al., 2018), we examined ROS level as well as apoptosis of IOs. As illustrated in Figure 6A, TNFα increased ROS level, an effect that was reversed by HYJJ. The swollen shape and dark lumen of IOs in the TNFα group were also rescued by HYJJ administration (30 ng/ml, 24 h). Moreover, the number of multi-buds IOs was considerably decreased after TNFα treatment, which was successfully reversed by HYJJ (Figure 6B). Western blotting results showed the increased apoptosis in the presence of TNFα was suppressed by HYJJ, as evidence by the protein expression of BAC and BCL-2 (Figure 6C). Altogether, HYJJ facilitates the re-establishment of the ISCs niche in an *ex-vivo* model of ulcerative colitis.

### Huiyangjiuji Decoction Suppressed Inflammation and Contributed to Mucosal Healing

Mφs, antigen-presenting cells with high plasticity linking the innate and adaptive immune system, transit to the pro-inflammatory phenotype (M1 Mφs) and secret interleukins to amplify the inflammation processes, such as IL-2 and IL-12, as well as IL-10. Nevertheless, Mφs facilitate wound-healing when they act as guardians of the homeostasis and present M2 phenotype.

**FIGURE 7:**
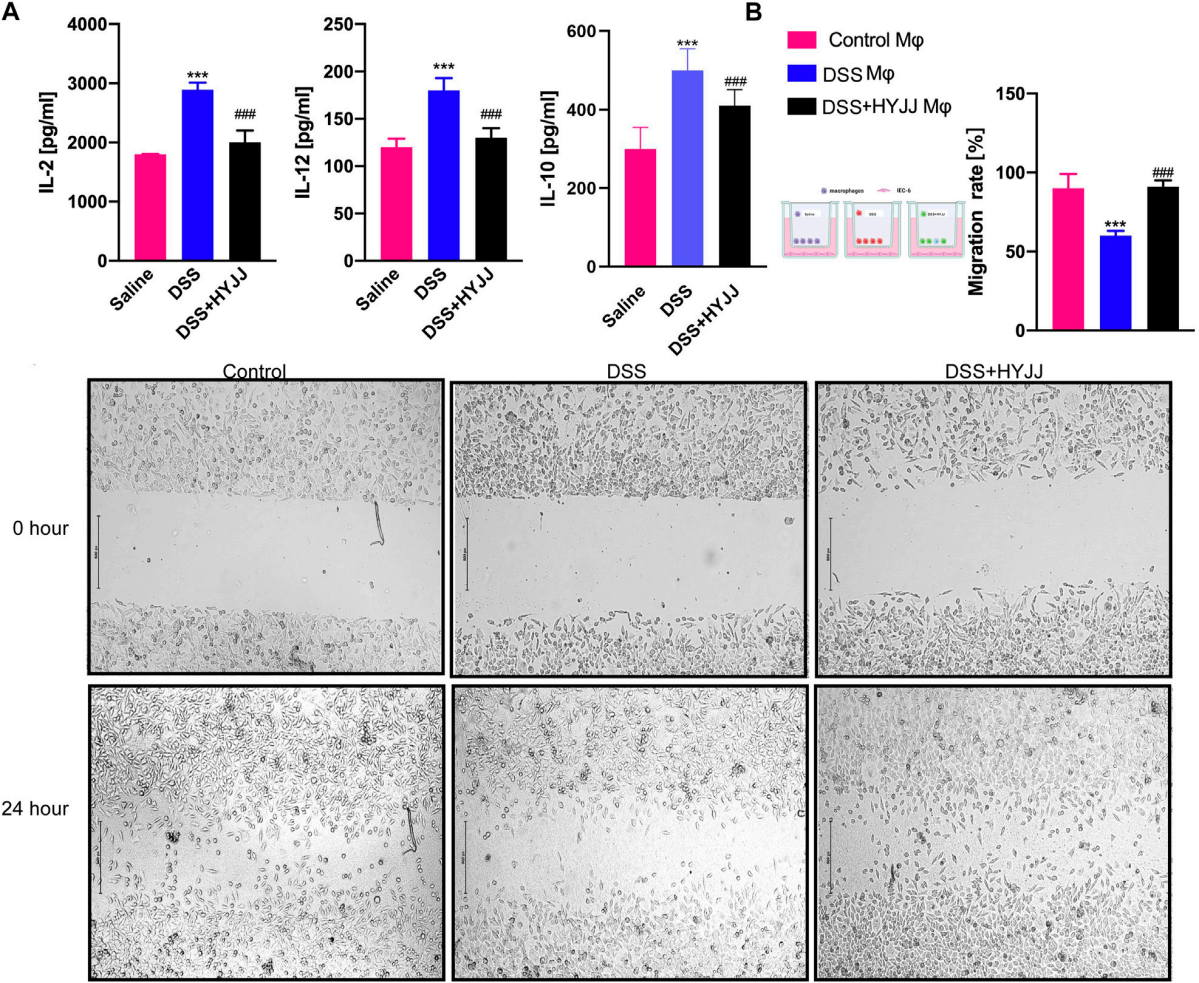
The roles of HYJJ in the inflammatory status.**(A)** Bar charts (*n* = 5) illustrating the levels of IL-2, IL-10 and IL-12 of UC mice with HYJJ treatment.**(B)** Scratch assay showing the migration capacity of IEC-6 cells co-cultured with macrophages from UC mice with HYJJ treatment. ***(*p* < 0.001) indicate statistically significant difference from saline group, ###(*p* < 0.001) indicate statistically significant difference from UC group.

ELISA confirmed that HYJJ administration inhibited the levels of IL-2, IL-10 as well as IL-12 in the serum collected from mice undergoing experimental colitis (Figure 7A). Moreover, We established a co-culture system by Mφs and IEC-6 cells to mimic the inflammatory microenvironment around epithelial cells. Firstly, we isolated peritoneal Mφs from HYJJ-DSS mice and co-cultured these Mφs with IEC-6 cells for 24 h. A scratch assay showed that Mφs from DSS mice hampered the migration capacity of IEC-6 cells, but no detrimental role of HYJJ-DSS Mφs was observed (Figure 7B). Altogether, HYJJ might inhibit M1 Mφs transition and hence suppressing the pro-inflammatory interleukins levels in DSS-induced colitis mice.

## Discussion

Large-scale functional genomics findings in various disease models have proposed that single-gene knockouts convey minimal information on phenotype. Only 19% of genes are crucial across model organisms, and most single-gene manipulations are likely to be involved in the prevalence rate under pathological stimuli (Hopkins, 2008). Therefore, the philosophy “one gene, one drug, one disease” of drug design has been challenged over the decades. Emerging network pharmacology has shed light on TCM and offered a practical approach to elucidate the pharmacological mechanisms of numerous compounds. Toxicity and efficacy account for the major sources of attrition in TCM therapy and have always aroused general concerns. In particular, inadequate animal *in-vivo* animal experiments and unknown pharmacological mechanisms lie at the heart of the query.

HYJJ decoction encompasses ten herbs, and the combination of ACD, ZR and GF has been introduced as Si-Ni decoction carrying on a potential anti-inflammatory effect on the treatment of septic and shock sepsis (Chen et al., 2011; Brislinger et al., 2018; Zeng et al., 2019). However, the therapeutic role of HYJJ in UC has not yet been elucidated. UC is the subtype of IBD and leads to digestive disorders, inflammation and even UC-CRC. In the present study, our *in vivo* findings showed that HYJJ effectively alleviated the progression of experimental colitis, and explored the pharmacological mechanism utilizing network pharmacology analysis. By integrating UC-associated genes from five databases and obtaining the active components of HYJJ in the TCMSP database, 218 target genes were identified. ΚΕGG analysis demonstrated the enrichment of hub genes in cancer as well as infection pathways, which corroborated the anti-inflammatory role of HYJJ. In the formula, combating inflammation was the principal target accomplished by eight herbs, such as ACD, ZR, GF, PGM (Jin et al., 2020), PB (Tian et al., 2020), SCF (Li et al., 2020; Li et al., 2021), PCW (Zhang et al., 2018) as well as CC (Csikos et al., 2020). In addition to inflammation, the recovery of gut motility and orchestration of the gut innervation were also underlined. On the one hand, adrenergic (Nezi et al., 2000; Furlan et al., 2006) and cholinergic (Mogilevski et al., 2019) systems work synergistically to maintain the homeostasis of the gastrointestinal tract, and our KEGG analysis demonstrated the regulatory role of PB in adrenergic and catecholamine activity, and CR in endocrine resistance. On the other hand, given the dysmotility caused by the inflammatory mediators and neural circuitry during UC progression, HYJJ included ACD, AMK, and PSW to improve the contraction of gut muscles. Consistently, HYJJ decoction took the brain-gut axis into consideration (Teratani et al., 2020) and selected compounds able to influence neurotransmitter activity. GO and KEGG analysis showed that AMK, PSW and SCF target genes were not only enriched in neurological pathways, including neuroactive ligand-receptor interaction and neurotransmitter receptor activities, but also contributed to digestive functions, such as protein digestion and absorption, as well as salivary secretion. Many of these effects were seemingly rather indirectly combating secretory diarrhea frequently occurring during IBD (Schiller, 1999), than directly interacting with the progression of IBD (Jonge et al., 2020). Additionally, given the high fiber in the components of HYJJ decoction and the improved gut health following ingestion of plant-based constituents (Carvalhana et al., 2012; Alferink et al., 2020), the intrinsic nature of these herb compounds was also beneficial for the gut recovery. Collectively, network pharmacological analysis demonstrated that HYJJ provided a potential therapeutic strategy for UC handling by suppressing inflammation, restoring the homeostasis of hormones and innervation, and facilitating gut motility and digestion.

In a bid to corroborate the findings above, DLD-1 cells were treated with the critical components of HYJJ and performed RNA-sequencing. In line with the results of network pharmacological analysis, HYJJ down-regulated UC-related inflammatory pathways, such as IBD and TNF, NOD-like and Toll-like receptor signaling pathways, and facilitated salivary and pancreatic secretion, glutamatergic and dopaminergic synapse, fat digestion and absorption, all of which were mentioned in network analysis about the individual roles of HYJJ. Most interestingly, the inevitable concerns about the toxicity of HYJJ were also clearly addressed. First, the primary active components in the decoction were acid chemicals, especially GF, and might hamper the mucosa, and CR could counteract and neutralize the acid irritation (Figure 3B). Secondly, the detrimental role of ACD for gestation has been proposed in Ben Cao Gang Mu, the most complete medical monograph written in the history of traditional Chinese medicine by LI Shi-Zhen, and KEGG analysis also confirmed the association of ACD and SCF with the maternal process involved in female pregnancy. Given the reported protective role of SCF in pregnancy (Panossian and Wikman, 2008), we proposed that the toxicity of ACD might be neutralized by SCF, which needs further experimental validations *in vivo* and *in vitro*.

Our *ex-vivo* experiments further demonstrated the positive role of HYJJ for mucosal healing and anti-inflammation. IO is a primary culture of intestinal stem cells and represents the dynamic progression of mucosal recovery, providing a specific powerful tool to mimic the interplay between intestinal stem cells and immune cells and mostly suitable for investigating IBD (Yin et al., 2018; Deuring et al., 2019). Stem cells are in a quiescent state to maintain a long-lived capacity in non-inflammatory settings, and enter into a proliferative state upon stimulations including excessive ROS, thereby undergoing apoptosis (Tothova et al., 2007; Zhang et al., 2018). We found the exacerbated ROS and apoptosis of IOs could be rescued after HYJJ treatment, which suggested that HYJJ was beneficial for re-epithelization. Furthermore, the phenotypes of Mφs reflect the immune status inside the body and exert distinct functions. In the case of inflammation, M1 Mφs secret pro-interleukins and amplify immune responses by antigen-presenting capacity. However, M2 Mφs act as safe guardians for homeostasis and wound-healing. We therefore isolated peritoneal Mφs from HYJJ-UC mice and co-cultured with IEC-6 cells, aiming to mimic the immunological environment of the ISCs niche. As a result, HYJJ treatment decreased the levels of pro-inflammatory interleukins in the serum of UC mice, and Mφs from HYJJ-treated mice did not compromise the migration of IECs, pointing out the anti-inflammatory role of HYJJ in UC.

## Conclusion

By using a computational systems pharmacology approach and experimental evidence, we concluded that HYJJ alleviates experimental colitis progression by improving epithelial reconstitution and inhibiting pro-inflammatory response.

## Data Availability

The original contributions presented in the study are included in the article/Supplementary Material data as well as in SRA database (PRJNA724825).

## Abbreviations

IBD, Inflammatory bowel disease; IOs, intestinal organoids; Mφs, Macrophages; HYJJ, *Huiyangjiuji* decoction; ISCs, intestinal stem cells; TCM, Traditional Chinese Medicine; PPIs, Protein-protein interactions; MOS, mitochondrial stress; UC, Ulcerative colitis.
