# Supplementary figures and images for: Network Pharmacology-Based Validation of the Efficacy of Huiyangjiuji Decoction in the Treatment of Experimental Colitis
Source: Front Pharmacol. 2021 May 28;12:666432. doi: 10.3389/fphar.2021.666432 (PMC8193934; doi:10.3389/fphar.2021.666432)

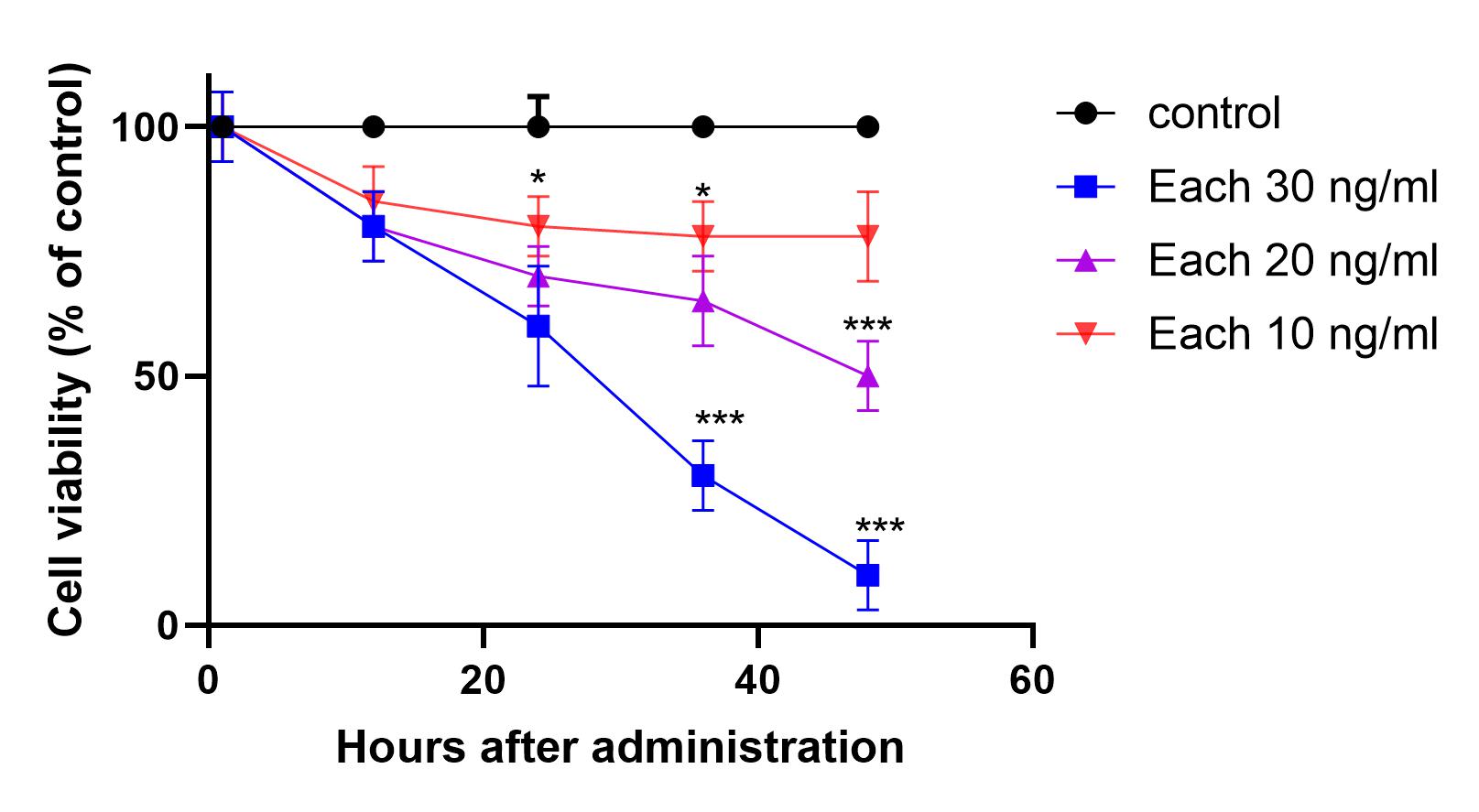

Supplement: Supplementary file 2 [file Image3.JPEG]

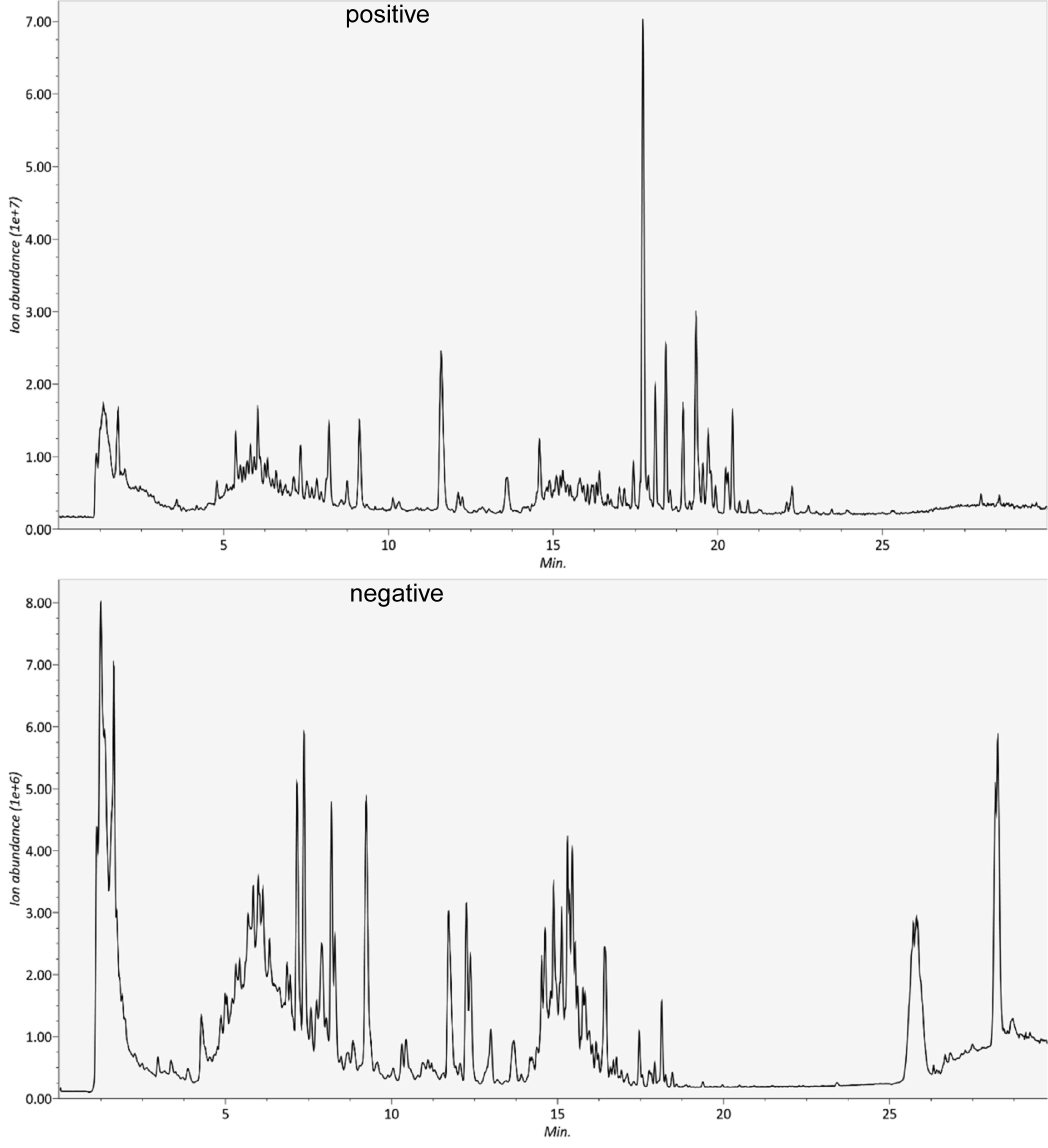

Supplement: Supplementary file 4 [file Image1.JPEG]

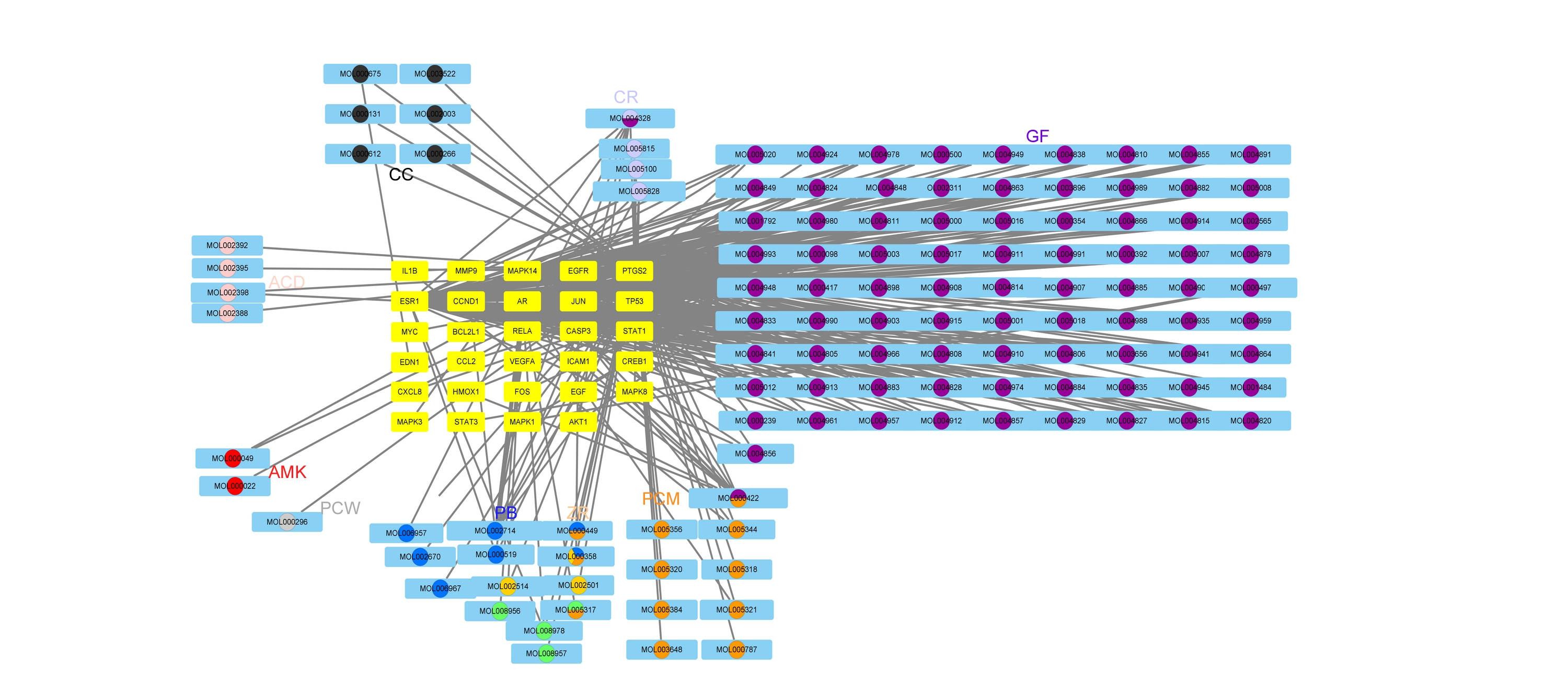

Supplement: Supplementary file 5 [file Image2.JPEG]
